# Prognostic significance of the C-reactive protein–albumin–lymphocyte index and the pan-immune-inflammation value in ischemic and hemorrhagic stroke: a comparative analysis of subtypes

**DOI:** 10.3389/fneur.2025.1724077

**Published:** 2026-01-12

**Authors:** Canan Şahin, Yahya Şahin

**Affiliations:** Department of Emergency Medicine, Ahi Evran University Faculty of Medicine, Kırşehir, Türkiye

**Keywords:** biomarker, CALLY index, inflammation, PIV, prognosis, stroke subtypes

## Abstract

**Background:**

Cerebrovascular events are major causes of global mortality and disability. Inflammation, immune response, and nutritional status play crucial roles in stroke pathophysiology. The C-reactive protein–albumin–lymphocyte (CALLY) index and the pan-immune-inflammation value (PIV) are novel composite biomarkers reflecting these mechanisms. This study compared their prognostic performance between ischemic and hemorrhagic stroke.

**Methods:**

This retrospective, single-center cohort included 357 patients diagnosed with ischemic or hemorrhagic stroke between January 2020 and December 2024. Demographic, clinical, and laboratory data were retrieved from electronic records. Indices including CALLY, PIV, HALP, SII, and MII were calculated from blood samples obtained within 24 h of admission. Group comparisons, multivariate logistic regression, and receiver operating characteristic (ROC) analyses identified independent predictors and diagnostic performance.

**Results:**

Among 357 patients (mean age 67 ± 14 years; 56.6% ischemic, 43.4% hemorrhagic), the CALLY index was significantly higher in hemorrhagic cases (*p* < 0.001). Multivariate analysis identified CALLY (odds ratio = 1.255), PIV (odds ratio = 1.001), and HALP (odds ratio = 1.258) as independent predictors (all *p* < 0.001). CALLY showed the highest discriminative ability (area under the ROC curve = 0.756). In hemorrhagic stroke, lower CALLY values were associated with in-hospital mortality (AUC = 0.646, *p* = 0.002).

**Conclusion:**

The CALLY index demonstrated superior prognostic value compared with other inflammatory markers and may serve as a simple, low-cost tool for early stroke risk assessment. Prospective, multicenter studies are warranted to validate these findings and to establish standardized cutoff values.

## 1 Introduction

Cerebrovascular events (CVE) remain one of the predominant causes of mortality and morbidity worldwide. According to the 2019 Global Burden of Disease report, approximately 12.2 million new stroke cases and 6.55 million stroke-related deaths were documented, establishing stroke as the second leading cause of death on a global scale (1). The considerable mortality and long-term disability resulting from stroke deteriorate patients’ quality of life. These consequences also impose an extensive and escalating economic burden on healthcare systems. These challenges are particularly pronounced in low- and middle-income countries, where disparities in rehabilitation services and long-term care underscore the urgent need to strengthen both primary and secondary prevention strategies.

Ischemic stroke and hemorrhagic stroke constitute the two principal subtypes of cerebrovascular events. Ischemic strokes account for approximately 70–80% of all cases and are typically associated with atherosclerosis, cardioembolism, or small-vessel occlusion, whereas hemorrhagic strokes are less common but carry a markedly higher risk of mortality. This clinical distinction underscores the critical importance of early diagnosis and accurate risk stratification (2). Differentiating stroke subtypes guides acute therapeutic interventions. It also informs intensive care management and secondary prevention planning. The evaluation of biochemical biomarkers in conjunction with clinical scoring systems during the acute phase may enhance treatment decision-making and improve the prediction of hemorrhagic complications.

In recent years, the role of inflammation in the pathophysiology of stroke has become increasingly evident. Low-cost, blood count–based indices [Neutrophil-to-Lymphocyte Ratio (NLR), Platelet-to-Lymphocyte Ratio (PLR), and Systemic Immune-Inflammation Index (SII)] have been associated with early neurological deterioration and long-term outcomes in acute ischemic stroke (3–5). Meta-analyses have demonstrated that elevated SII levels are correlated with higher mortality and poor prognosis (4, 6). Composite indices such as the Multi-Inflammatory Index (MII-1, MII-2, and MII-3) have also been linked to increased mortality risk (7). These hematologic markers reflect systemic inflammatory burden through the interplay of neutrophils, monocytes, lymphocytes, and platelets.

The Pan-Immune Inflammation Value (PIV) is a novel composite parameter that holistically reflects the balance among neutrophils, monocytes, platelets, and lymphocytes. PIV has been independently associated with unfavorable functional outcomes following intravenous thrombolysis and mechanical thrombectomy, and large cohort studies have demonstrated its predictive power for both stroke risk and mortality (8–11). Its easy derivation from routine complete blood counts facilitates integration into clinical practice; however, it is recommended that PIV be evaluated in conjunction with the National Institutes of Health Stroke Scale (NIHSS) and comorbidity burden within multivariable models.

The C-reactive protein–albumin–lymphocyte (CALLY) index simultaneously evaluates inflammation, nutritional status, and immune response. Low CALLY values have been associated with poor functional outcomes and hemorrhagic transformation, whereas data from the NHANES cohort indicate that higher CALLY levels are linked to a reduced risk of stroke (12, 13). In patients with acute ischemic stroke undergoing endovascular treatment, a low CALLY index serves as an independent predictor of unfavorable prognosis (12, 14, 15). Moreover, the association of CALLY with mortality in patients with STEMI, sepsis, and critical illness supports its biological plausibility (16–19). CRP reflects systemic inflammation, albumin represents the inflammation–malnutrition axis, and lymphocyte count indicates immune competence, collectively providing an integrated perspective on post-stroke recovery potential. CALLY and PIV represent complementary dimensions of the inflammatory–nutritional–immune axis, suggesting that their combined assessment may provide a more comprehensive prognostic signal in stroke than either marker alone. Similarly, the hemoglobin–albumin–lymphocyte–platelet (HALP) score is a composite biomarker that integrates hematologic, inflammatory, and nutritional parameters to reflect systemic immune-inflammatory status. Low HALP levels have been correlated with adverse outcomes and increased mortality across various cardiovascular and neurological disorders (20).

The aim of this study was to comparatively evaluate the prognostic performance of the CALLY index and the Pan-Immune Inflammation Value (PIV) in patients with ischemic and hemorrhagic stroke, thereby addressing the current gap in the literature regarding their combined assessment.

In conclusion, both the Pan-Immune Inflammation Value (PIV) and the C-reactive protein–albumin–lymphocyte (CALLY) index serve as robust prognostic biomarkers in both ischemic and hemorrhagic strokes by integrating inflammatory, immunological, and nutritional axes. Since the majority of previous studies have predominantly focused on a single stroke subtype, the comparative approach adopted in this study aims to provide direct translational relevance to clinical practice. Looking forward, standardizing the temporal dynamics and treatment-response monitoring potential of these indices through multicenter validation studies will facilitate their integration into clinical decision-support systems.

## 2 Materials and methods

This retrospective, single-center cohort study was conducted among patients who presented to the Emergency Department of Ahi Evran University Faculty of Medicine Hospital with a diagnosis of stroke between January 1, 2020, and December 31, 2024. Ethical approval was obtained from the Kırşehir Ahi Evran University Health Sciences Scientific Research Ethics Committee (Decision No: 2025-12/153; Date: 22.07.2025), and the study was carried out in accordance with the principles of the Declaration of Helsinki. Since this was a retrospective study based on previously recorded and anonymized patient data, the study period naturally preceded the date of ethical approval. The ethics committee granted approval retrospectively, which is in accordance with institutional guidelines for retrospective research. Due to its retrospective design—based solely on previously recorded and anonymized clinical data—and the minimal risk posed to participants, the Ethics Committee exempted the study from the requirement of obtaining written informed consent. Patients aged 18 years or older with a diagnosis of ischemic or hemorrhagic stroke confirmed by computed tomography (CT) or magnetic resonance imaging (MRI) were included in the study, whereas those with hematologic malignancy, active infection, autoimmune disease, a history of corticosteroid or immunosuppressive drug use, recurrent stroke, or missing data in key variables were excluded from the analysis.

All data were retrospectively retrieved from the hospital’s electronic medical record system. In addition to demographic (age, sex, smoking status), clinical (hypertension, diabetes), and laboratory variables, inflammatory and immuno-nutritional indices were calculated, with details regarding formulas and measurement units provided in the section titled “Laboratory Measurements and Index Calculations.” Patients were classified into two groups—ischemic and hemorrhagic stroke—based on their diagnosis, and these groups were compared in terms of demographic, clinical, and laboratory characteristics as well as index values. Furthermore, the prognostic performance of these indices was evaluated between patients who survived and those who died during follow-up. All identifying information was anonymized, and strict adherence to confidentiality principles was maintained.

### 2.1 Laboratory measurements and index calculations

All hematological and biochemical parameters were obtained from the first blood samples collected within the initial 24 h following hospital admission. Cellular counts were standardized in units of 10^9^/L, albumin in g/dL, and C-reactive protein (CRP) in mg/L. When CRP values were reported in mg/dL, a conversion factor of 1 mg/dL = 10 mg/L was applied; likewise, cellular counts expressed in 10^3^/μL were considered equivalent to 10^9^/L. The indices were calculated as follows:

NLR = neutrophil/lymphocyte; PIV = (neutrophil × platelet × monocyte) / lymphocyte (all cellular counts expressed in 10^9^/L); CALLY = [albumin (g/dL)] / [CRP (mg/L) × NLR] × 10. The ×10 coefficient in the CALLY formula was applied as a scaling factor to enhance numerical readability without altering the clinical interpretation of values. In addition, the MII-1, MII-2, MII-3, Systemic Immune-Inflammation Index (SII), and Hemoglobin–Albumin–Lymphocyte–Platelet (HALP) indices were calculated using the standard formulas previously defined in the literature.

### 2.2 Statistics

Descriptive statistics for the variables were presented as n (%), or Median (Min–Max). The normality of continuous data was assessed using the Kolmogorov–Smirnov and Shapiro–Wilk tests. Depending on the fulfillment of normality assumptions, comparisons between two groups were performed using the Mann–Whitney U test. For categorical variables, analyses were conducted using the Pearson Chi-square test, and when necessary, the Continuity Correction (Yates) was applied, taking into account the number of categories and expected cell frequencies.

A multivariate logistic regression analysis was performed to identify indices that could serve as potential diagnostic markers in ischemic and hemorrhagic patient groups. Similarly, in the ischemic and hemorrhagic CVE subgroups, multivariate logistic regression was applied to determine index-based risk scores (MII-1, MII-2, MII-3, SII, HALP, PIV, and CALLY) predictive of mortality. The backward elimination method was employed to select the most appropriate predictive model. In both ischemic and hemorrhagic CVE groups, the discriminative performance of the probability (risk) scores derived from the final logistic models in predicting mortality was evaluated using Receiver Operating Characteristic (ROC) curve analysis. The level of discrimination was quantified by calculating the area under the curve (AUC) along with corresponding 95% confidence intervals. To account for multiple comparisons in ROC analyses, Bonferroni correction was applied based on the number of indices evaluated. Both unadjusted and adjusted *p*-values were examined to ensure robustness of discrimination metrics.

All statistical analyses were conducted using IBM SPSS Statistics for Windows, Version 29.0 (IBM Corp., Armonk, NY, USA).

## 3 Results

A total of 357 patients were included in the study, of whom 155 (43.4%) were diagnosed with hemorrhagic and 202 (56.6%) with ischemic cerebrovascular events (CVE). The distribution of demographic and clinical characteristics is presented in Table 1. The mean age was significantly higher in the ischemic CVE group (*p* < 0.001). Regarding sex distribution, a male predominance was observed among hemorrhagic cases (69.7%), whereas a higher proportion of females (49.0%) was identified in the ischemic group (*p* < 0.001). The prevalence of hypertension and diabetes mellitus was significantly greater in the ischemic group (both *p* < 0.001). No significant difference was observed between the groups in terms of coronary artery disease (*p* = 0.301). The length of hospital stay was significantly longer in the ischemic group (*p* < 0.001). Mortality rates were comparable between the two groups (*p* = 0.149).

**Table 1:** Comparison of demographic and clinical characteristics of cerebrovascular event (CVE) cases by subtype.

Variable	Hemorrhagic (*n* = 155)	İschemic (*n* = 202)	*p*-value
Sex, *n* (%)			
Male	108 (69.7)	103 (51.0)	< 0.001
Female	47 (30.3)	99 (49.0)	
Age, Median (Min–Max)	59.0 (18–97)	72.0 (21–97)	< 0.001
Length of stay, Median (Min–Max)	3.0 (0–34)	5.0 (0–18)	< 0.001
HT, *n* (%)			
Absent	75 (43.1)	37 (18.3)	< 0.001
Present	99 (56.9)	165 (81.7)	
DM, *n* (%)			
Absent	145 (83.3)	112 (55.4)	< 0.001
Present	29 (16.7)	90 (44.6)	
CAD, *n* (%)			
Absent	129 (74.1)	140 (69.3)	0.301
Present	45 (25.9)	62 (30.7)	
Mortality, *n* (%)			
Alive	123 (70.7)	156 (77.2)	0.149
Deceased	51 (29.3)	46 (22.8)	

HT, hypertension; DM, diabetes mellitus; CAD, coronary artery disease.

The comparison of inflammatory indices is presented in Table 2. The hemorrhagic CVE group demonstrated significantly higher values of MII-1, MII-2, MII-3, HALP, and PIV (all *p* < 0.05). Conversely, the CALLY index was significantly higher in the hemorrhagic group (*p* < 0.001). No significant difference was observed between the groups in terms of SII values. These findings suggest that the inflammatory burden is markedly elevated in hemorrhagic CVE, whereas in ischemic CVE, nutritional and immune response components appear to play a more prominent role alongside inflammation.

**Table 2:** Comparison of inflammatory indices between ischemic and hemorrhagic cerebrovascular event (CVE) cases.

Inflammatory index	Hemorrhagic (Median [Min–Max])	Ischemic (Median [Min–Max])	*p*-value
MII-1	80.12 (2.31–3264.78)	25.53 (0.43–774.74)	<0.001
MII-2	2543.83 (104.12–81843.78)	980.52 (27.90–18401.58)	<0.001
MII-3	18364.0 (636.22–1090438.17)	6157.74 (125.57–192136.05)	<0.001
SII	804.21 (99.98–8136.58)	661.54 (161.56–6445.0)	0.194
HALP	5.06 (0.79–24.26)	4.32 (0.69–24.26)	0.002
PIV	556.36 (71.98–16632.26)	409.01 (74.98–3824.33)	0.010
CALLY	4.05 (0.16–87.30)	0.99 (0.03–42.0)	<0.001

MII-1, multi-inflammatory index-1; MII-2, multi-inflammatory index-2; MII-3, multi-inflammatory index-3; SII, Systemic Immune–Inflammation Index; HALP, hemoglobin–albumin–lymphocyte-platelet index; PIV, pan-immune–inflammation value; CALLY, C-reactive protein–albumin–lymphocyte index.

The results of the multivariate logistic regression analysis for predicting CVE subtypes are presented in Table 3. According to the findings, the CALLY (B = 0.227; *p* < 0.001; OR = 1.255; 95% CI: 1.162–1.354), PIV (B = 0.001; *p* < 0.001; OR = 1.001; 95% CI: 1.000–1.001), and HALP (B = 0.229; *p* < 0.001; OR = 1.258; 95% CI: 1.140–1.387) indices were identified as independent and statistically significant predictors of the hemorrhagic subtype of CVE. The overall classification accuracy of the model was 77.7%, indicating that the combined use of these three indices provides a robust biomarker profile for differentiating cerebrovascular event subtypes.

**Table 3:** Multivariate logistic regression analysis of inflammatory indices for predicting the subtype of cerebrovascular event (ischemic vs. hemorrhagic).

Variable	B	SE	Wald	*p*	OR	95% Cl (lower-upper)
PIV	0.001	0.003	15.327	<0.001	1.001	1.000–1.001
CALLY	0.227	0.039	33.675	<0.001	1.255	1.162–1.354
HALP	0.229	0.050	21.023	<0.001	1.258	1.140–1.387

PIV, pan-immune–inflammation value; CALLY, C-reactive protein–albumin–lymphocyte index; HALP, hemoglobin–albumin–lymphocyte-platelet index; B, regression coefficient; SE, standard error; Wald, Wald chi-square statistic used to test the significance of individual predictors; *p*, probability value indicating statistical significance; OR, odds ratio; 95% CI, 95% confidence interval (lower–upper limits).

**Figure 1:**
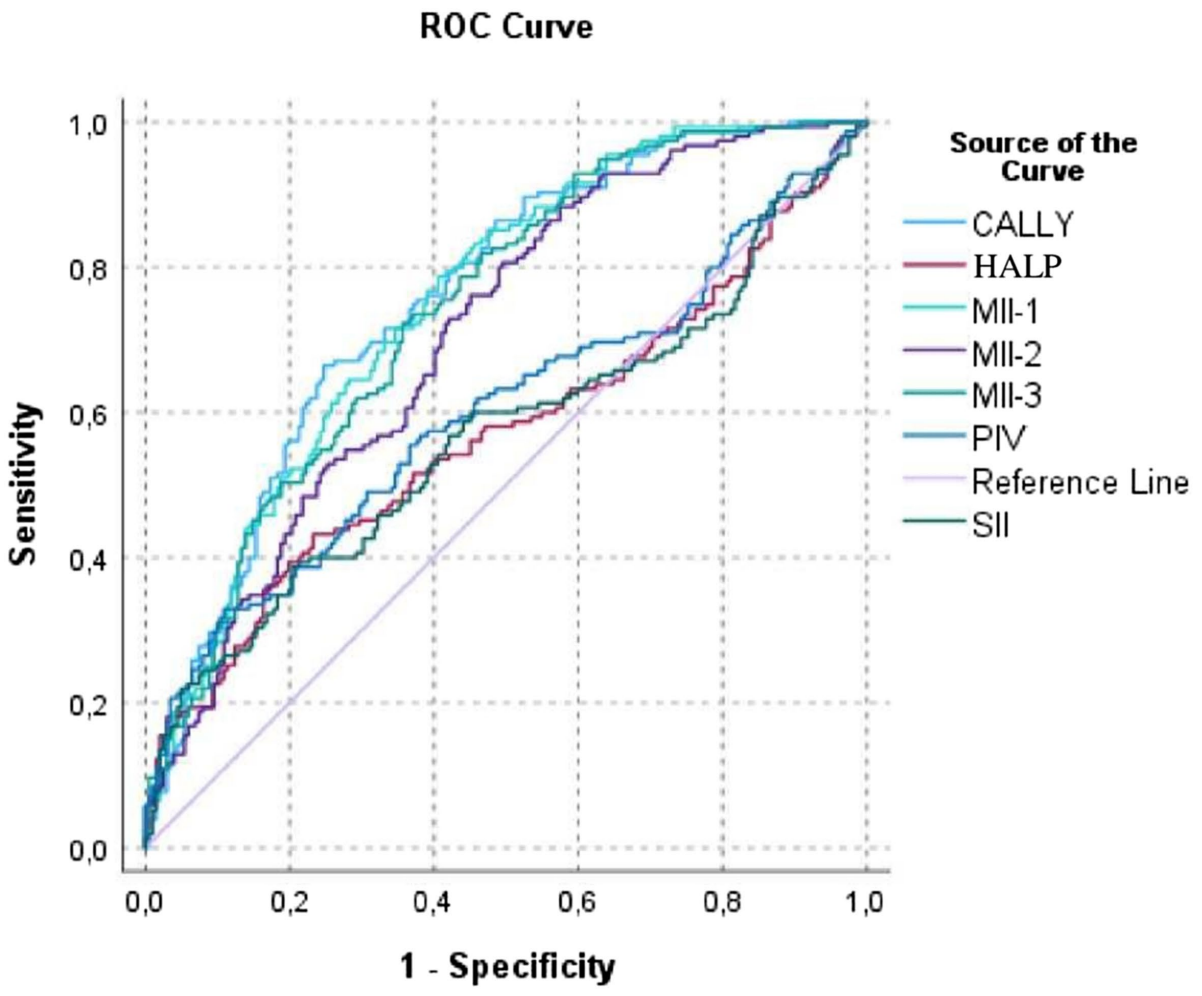
Receiver operating characteristic (ROC) curves of inflammatory indices for differentiating ischemic and hemorrhagic cerebrovascular events.

The results of the ROC analysis are presented in Table 4 and Figure 1. According to the findings, the CALLY (AUC = 0.756, *p* < 0.001) and MII-1 (AUC = 0.750, *p* < 0.001) indices exhibited the highest discriminative power in distinguishing CVE subtypes. The MII-2 and MII-3 indices demonstrated moderate discriminative performance, whereas the SII, PIV, and HALP indices showed limited predictive contribution. The Kolmogorov–Smirnov (K–S) statistics for the CALLY and MII-1 models were 0.417 and 0.381, respectively, indicating that these indices achieved the highest classification accuracy. The ROC curves (Figure 1) further illustrate that these two indices most effectively differentiate hemorrhagic and ischemic cases.

**Table 4:** ROC analysis results of inflammatory indices for differentiating the subtypes of cerebrovascular events.

Index	AUC	Std. error	*p*	95% CI	Cut-off	Max. K–S
CALLY	0.756	0.025	<0.001	0.707	2.25	0.417
MII-1	0.750	0.025	<0.001	0.701	35.59	0.381
MII-2	0.704	0.027	<0.001	0.651	993.91	0.311
MII-3	0.740	0.026	<0.001	0.690	9885.00	0.366
SII	0.566	0.032	0.038	0.504	1222.86	0.184
PIV	0.599	0.031	0.001	0.538	1114.48	0.220
HALP	0.570	0.032	0.028	0.508	5.91	0.200

CALLY, C-reactive protein–albumin–lymphocyte index; MII-1, multi-inflammatory index-1; MII-2, multi-inflammatory index-2; MII-3, multi-inflammatory index-3; SII, systemic immune–inflammation index; HALP, hemoglobin–albumin–lymphocyte-platelet index; PIV, pan-immune–inflammation value; ROC (receiver operating characteristic): Duyarlılık–(1 − özgüllük) uzayındaki eğri; bir testin ayırt ediciliğini gösterir, AUC (Area Under the ROC Curve): ROC eğrisi altındaki alan; ayırt edicilik ölçütü (0.5 = ayrım yok, 0.7–0.8 = orta/iyi, 0.8–0.9 = çok iyi, >0.9 = mükemmel), Std. error (standard error): AUC tahmininin örnekleme hatası; belirsizliğin düzeyini gösterir, *p* (*p*-value): AUC’nin 0.5’ten (şansa bağlı sınıflama) istatistiksel olarak farklı olup olmadığını test eden olasılık değeri (genellikle DeLong testi veya benzeri), 95% CI (95% confidence interval): AUC için %95 güven aralığı; gerçek AUC’nin bu aralıkta bulunması beklenir, Cut-off (optimal threshold): Duyarlılık ile özgüllük arasında en uygun dengeyi sağlayan eşik değer (çoğunlukla maksimum K–S ya da eşdeğeri Youden J = Duyarlılık + Özgüllük − 1 ölçütüne göre seçilir), Max. K–S (Maximum Kolmogorov–Smirnov Statistic): Pozitif ve negatif grupların kümülatif dağılımları arasındaki en büyük fark; ROC bağlamında Duyarlılık + Özgüllük − 1 ile eşdeğerdir (0–1 arası, 1 mükemmel ayrım gücü).

Mortality analyses yielded significant results only within the hemorrhagic CVE group. As shown in Table 5, the CALLY index was the only parameter that demonstrated a statistically significant association with mortality (*p* = 0.006). CALLY levels were significantly lower in patients who died. Median values were 2.74 in non-survivors and 4.96 in survivors. Although MII-1 values were higher in non-survivors, this difference was not statistically significant (*p* = 0.011). No significant associations were observed between mortality and the other indices (MII-2, MII-3, SII, HALP, and PIV) (*p* > 0.05). Taken together, these results demonstrate that the CALLY index was the only inflammatory–nutritional biomarker reaching statistical significance in hemorrhagic CVE, underscoring its unique prognostic value for in-hospital mortality.

**Table 5:** Comparison of inflammatory indices according to mortality in hemorrhagic cerebrovascular event (CVE) cases.

Index	Alive (Median [Min–Max])	Deceased (Median [Min–Max])	*p*-value
MII-1	59.20 (2.31–3264.78)	114.07 (6.21–1643.06)	0.011
MII-2	1888.15 (104.12–81843.78)	3200.00 (335.10–46388.89)	0.085
MII-3	13963.25 (636.22–1090438.17)	28512.00 (2031.82–548780.56)	0.092
SII	779.60 (99.98–8136.58)	881.26 (130.17–5487.81)	0.798
HALP	5.30 (0.79–21.00)	4.72 (0.86–24.26)	0.380
PIV	542.93 (71.98–16632.26)	579.22 (77.95–4207.17)	0.991
CALLY	4.96 (0.16–38.59)	2.74 (0.23–24.84)	0.006

MII-1, multi-inflammatory index-1; MII-2, multi-inflammatory index-2; MII-3, multi-inflammatory index-3; SII, systemic immune–inflammation index; HALP, hemoglobin–albumin–lymphocyte-platelet index; PIV, pan-immune–inflammation value; CALLY, C-reactive protein–albumin–lymphocyte index.

**Figure 2:**
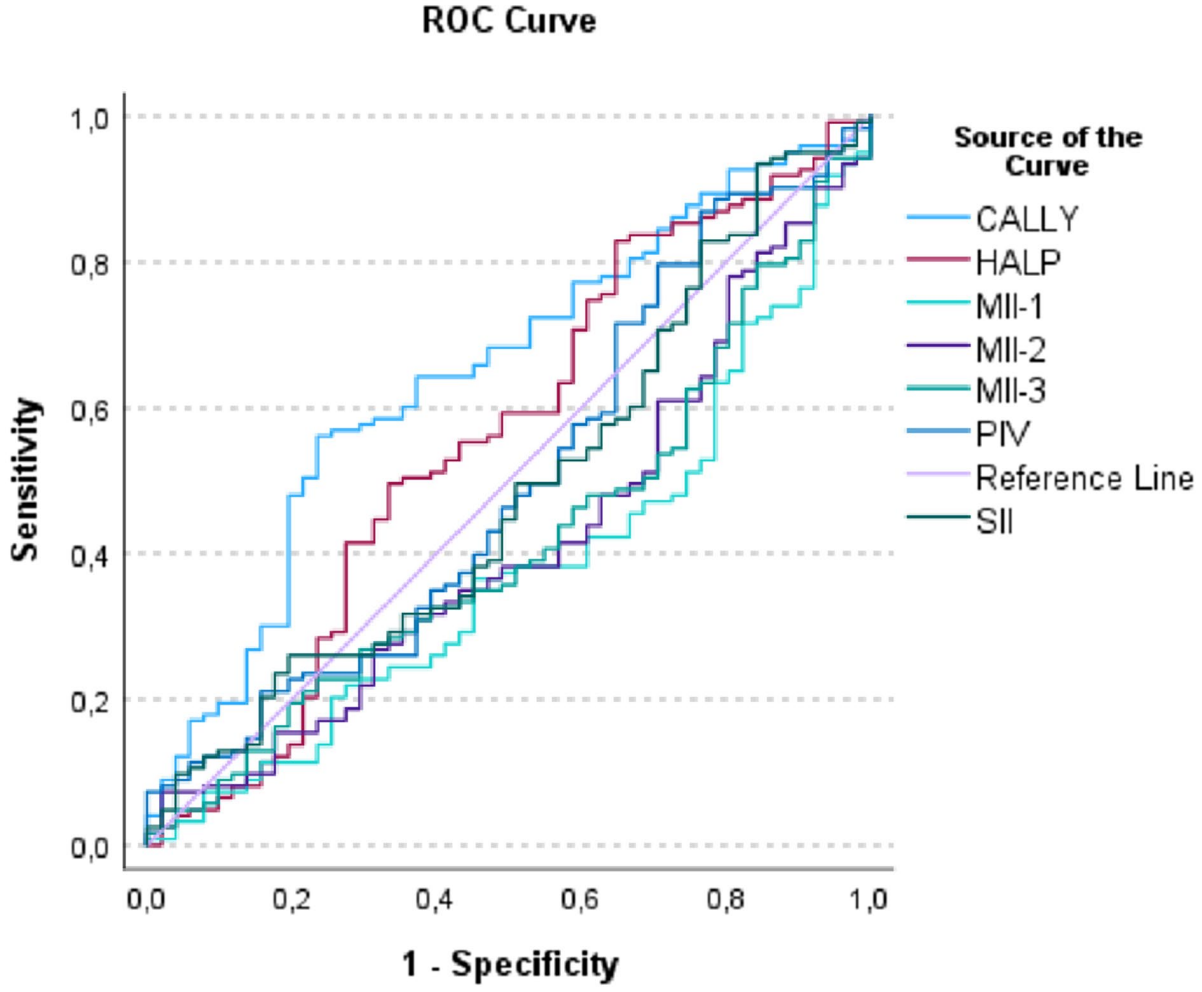
Receiver operating characteristic (ROC) curves of inflammatory indices for predicting mortality in hemorrhagic cerebrovascular event cases.

The results of the ROC analysis for predicting mortality in hemorrhagic CVE are presented in Table 6 and Figure 2. According to the analysis, the CALLY index emerged as the most statistically significant and clinically valuable biomarker for mortality prediction (AUC = 0.646, 95% CI: 0.557–0.735, *p* = 0.002). The AUC values of the other indices (MII-1, MII-2, MII-3, SII, HALP, and PIV) remained around 0.50, and their discriminative abilities did not reach statistical significance.

**Table 6:** ROC analysis of inflammatory indices for predicting mortality in hemorrhagic cerebrovascular event (CVE) cases.

Parameter	AUC	Std. error	*p*-value	95% CI	Gini	K–S	Cut-off
CALLY	0.646	0.045	0.002	0.557–0.735	0.292	0.326	0.0485
MII-1	0.378	0.045	0.011	0.289–0.467	−0.244	0.008	2453.92
MII-2	0.417	0.047	0.085	0.325–0.509	−0.166	0.054	17171.71
MII-3	0.419	0.046	0.092	0.328–0.510	−0.162	0.016	721257.29
SII	0.488	0.049	0.798	0.392–0.583	−0.025	0.092	248.48
HALP	0.556	0.050	0.244	0.457–0.655	0.112	0.182	0.2854
PIV	0.501	0.049	0.991	0.404–0.597	0.001	0.105	204.92

CALLY, C-reactive protein–albumin–lymphocyte index; MII-1, multi-inflammatory index-1; MII-2, multi-inflammatory index-2; MII-3, multi-inflammatory index-3; SII, systemic immune–inflammation index; HALP, hemoglobin–albumin–lymphocyte-platelet index; PIV, pan-immune–inflammation value; AUC, area under the curve, indicates the overall diagnostic performance of each index; Std. error, standard error, reflects the variability of the AUC estimate; *p*-value, statistical significance of the AUC value. 95% CI, 95% confidence interval for AUC; Gini, Gini coefficient, representing twice the deviation of AUC from random classification (AUC–0.5); K–S: Kolmogorov–Smirnov statistic, expressing the maximal difference between cumulative distribution functions of sensitivity and 1–specificity; Cut-off: the optimal threshold value for predicting mortality, determined by the maximum Youden or K–S index.

Sensitivity analyses stratified by major comorbidities (hypertension and diabetes mellitus) further supported the robustness of the CALLY index. The AUC values remained highly consistent across strata—0.643 vs. 0.652 in hypertensive and non-hypertensive patients, and 0.640 vs. 0.649 in diabetic and non-diabetic patients—demonstrating that comorbidity burden did not materially affect its prognostic performance.

Moreover, after applying Bonferroni correction for multiple comparisons (α_adjusted = 0.007), CALLY was the only biomarker that retained statistical significance. All other indices lost significance following adjustment, underscoring that CALLY is the sole mortality-related indicator with truly robust and reliable discriminative ability in patients with hemorrhagic stroke.

The ROC curve in Figure 2 illustrates that the CALLY index, by encompassing a larger area under the curve, demonstrated the highest predictive performance for mortality compared with the other parameters. These findings further reinforce that CALLY was the only biomarker with meaningful discriminative ability in hemorrhagic mortality prediction.

## 4 Discussion

In the pathophysiology of stroke, the interplay between inflammation, immune response, and nutritional status is now understood within a more integrated and biologically grounded framework. The CALLY index, which simultaneously reflects these axes, offers a practical, rapid, and clinically applicable biomarker approach for risk prediction. Previous studies have reported that lower CALLY levels increase the risk of stroke and provide prognostic superiority over the SII and SIRI indices (12), that higher CALLY values are associated with a reduced likelihood of stroke among hypertensive individuals (13), and that lower CALLY serves as an independent predictor of poor prognosis in patients undergoing endovascular thrombectomy (15). Moreover, its inverse association with mortality across diverse populations (21), marked protective effect on all-cause mortality in elderly cohorts (22), and ability to predict early mortality in critically ill patients (23) collectively support the generalizability of this biomarker. Consistent with these findings, the biological rationale underlying the CALLY index—comprising CRP as an indicator of the acute-phase response, albumin as a negative acute-phase reactant, and lymphocyte count as a marker of immune competence—strengthens the biological plausibility of the present study. The measurement of these parameters in the early phase using standardized protocols further enhances the reliability and clinical interpretability of the results.

Our study comparatively demonstrated the prognostic significance of the CALLY and PIV indices in both ischemic and hemorrhagic stroke. The observed differences between subtypes indicate that inflammatory and nutritional responses contribute distinctly to the underlying pathophysiological mechanisms. The combined assessment of these two indices may provide additional prognostic value in predicting mortality and functional outcomes. Consistent findings reported in oncological and population-based datasets (24, 25) further support the reproducibility and reliability of these biomarkers across diverse clinical cohorts. Consistent with data from the NHANES cohort indicating that higher CALLY levels are associated with a reduced risk of incident stroke, our findings demonstrate that higher CALLY values are linked to more favorable in-hospital outcomes, particularly among patients with hemorrhagic stroke. This parallel suggests that the protective role of preserved immuno-nutritional status may extend from primary prevention in the general population to prognostic stratification in acute cerebrovascular events. In clinical practice, integrating these indices into established risk assessment systems alongside conventional clinical scores—such as for initial risk stratification or reclassification during follow-up—has the potential to enhance the objectivity and predictive accuracy of clinical decision-making processes.

In ischemic stroke, hypoxia-induced cytokine release and microcirculatory dysfunction, and in hemorrhagic stroke, secondary inflammation and oxidative stress triggered by blood leakage into the parenchyma or meningeal spaces, represent converging clinical manifestations of systemic inflammation. Our findings indicate that higher CALLY levels in hemorrhagic stroke reflect the prognostic importance of immuno-nutritional components, whereas elevated PIV and MII values in hemorrhagic stroke appear to reinforce post-hemorrhagic tissue injury. Indeed, previous studies have reported that elevated PIV levels are associated with both short- and long-term mortality in non-traumatic subarachnoid hemorrhage (26). These distinct pathophysiological mechanisms suggest that temporal alterations in the cellular components represented by these indices (neutrophils, monocytes, platelets, and lymphocytes) may generate stroke subtype–specific immuno-inflammatory patterns. However, because imaging-based severity markers such as hematoma volume, ASPECTS, midline shift, or edema grade were not uniformly documented in the retrospective archive, these parameters could not be adjusted for and may contribute to residual confounding. This concept aligns with recent transcriptomic evidence demonstrating that neuroinflammatory signaling and immune-related gene pathways play critical roles in neurological injury. For example, a 2025 study investigating MC1R-mutant mice reported significant downregulation of immune-response pathways and reduced expression of angiogenin, a molecule involved in neuroprotection, oxidative-stress regulation, and vascular homeostasis (27). While not directly modeling stroke, this evidence supports the broader concept of immune–neuroinflammatory dysregulation and cellular stress responses in acute neurological injury. These mechanistic parallels support the biological plausibility of our findings, particularly the prognostic significance of indices such as CALLY that integrate inflammatory and immuno-nutritional dimensions.

PIV, as an integrative indicator of inflammation based on the neutrophil–monocyte–platelet–lymphocyte axis, has been associated with poor clinical outcomes following thrombolysis and thrombectomy (8, 9). Nevertheless, some studies have suggested that the standalone prognostic accuracy of PIV may be limited and that its combination with other inflammatory indices could enhance predictive performance (28). Its association with all-cause mortality in cardiometabolic and oncological populations (24, 25), as well as its identification as an independent risk marker in the intensive care management of severe neurological conditions (26), further strengthens the clinical applicability of PIV.

The prominence of the CALLY index as the strongest mortality-associated marker in hemorrhagic stroke suggests that the inflammation–hypoproteinemia–immune deficiency axis may adversely influence prognosis. In hemorrhagic stroke, acute vessel rupture and subsequent hematoma expansion are tightly linked to systemic and local inflammatory activation. CRP, as a key acute-phase reactant, has been implicated in endothelial dysfunction and vascular wall instability, whereas hypoalbuminemia and lymphopenia reflect impaired antioxidant capacity, disruption of capillary integrity, and reduced cellular immune competence. Accordingly, a low CALLY score—combining elevated CRP with reduced albumin and lymphocyte counts—may capture a particularly harmful immuno-nutritional profile that amplifies secondary injury after intracranial bleeding, which could explain its stronger prognostic performance in hemorrhagic stroke compared with ischemic events. The previously reported inverse association between CALLY and mortality across various disease groups further supports this interpretation (21). Its inverse relationship with both short- and long-term adverse cardiovascular events reinforces the clinical relevance of CALLY within the continuum of vascular pathology (16, 29). Accordingly, prospective evaluation of whether targeted interventions—such as nutritional optimization and infection control—can improve clinical outcomes in phenotypes characterized by low CALLY levels represents a clinically meaningful avenue for future research.

HALP, SII, and MII series also exhibit significant associations with prognosis. HALP reflects the balance between nutritional and inflammatory status, whereas SII represents systemic inflammatory activity (5, 30). The MII-1, MII-2, and MII-3 indices, which incorporate CRP, have been reported as significant predictors of mortality in acute ischemic stroke (7, 31, 32). These composite indicators have been shown to provide greater predictive power when used in conjunction with NIHSS scores and subtype-specific clinical variables (33). In clinical application, considering variable interrelations and the potential risk of overfitting within predictive models, as well as adopting dynamic approaches that allow risk monitoring over time, may contribute to obtaining more reliable and generalizable outcomes.

After Bonferroni correction, CALLY remained the strongest discriminator among all indices. The optimal cutoff for CALLY (2.25) represents a clinically relevant threshold, balancing sensitivity and specificity for differentiating ischemic and hemorrhagic stroke. Although modest, this cutoff identifies patients with a more pronounced inflammatory–nutritional imbalance. Clinically, values below this threshold may indicate a higher probability of ischemic pathology and warrant closer observation. Conversely, indices that lost statistical significance after adjustment (SII and HALP) appear less reliable for subtype discrimination in routine practice.

From a clinical perspective, the optimal CALLY cut-off identified in ROC analyses (0.0485) captures patients with pronounced inflammatory and nutritional compromise. Although its AUC (0.646) indicates modest discrimination, this threshold prioritizes sensitivity, facilitating early identification of hemorrhagic stroke patients at increased mortality risk. Patients presenting with CALLY values below this cut-off may benefit from intensified monitoring, nutritional optimization, and individualized management strategies. Importantly, after Bonferroni correction, CALLY remained the only statistically robust predictor of mortality, further supporting its clinical relevance as an accessible, low-cost prognostic biomarker. The persistence of CALLY as the sole significant index after Bonferroni correction emphasizes that its prognostic utility is not a statistical artifact of multiple testing but reflects a genuine, biologically coherent signal. This finding reinforces the pivotal role of the inflammation–nutrition–immune axis in determining outcomes in hemorrhagic stroke.

In conclusion, composite indices that holistically reflect the inflammation–immune response–nutrition axis exhibit distinct prognostic profiles across different stroke subtypes. CALLY demonstrated discriminative value in ischemic cases and significant predictive capacity for mortality in hemorrhagic cases; these biomarkers appear to be practical, cost-effective, and easily integrable tools for early clinical decision-making. Future studies should prioritize multicenter and prospective designs to validate multibiomarker panels and integrated risk models according to treatment modalities (21, 24–26, 33, 34). Furthermore, promoting the implementation of electronically compatible, automatically computable indices and conducting independent cohort validations across diverse demographic and cardiometabolic profiles will enhance the long-term sustainability of their clinical utility.

## 5 Conclusion and future perspectives

This study demonstrates that the C-reactive protein–albumin–lymphocyte (CALLY) index and the Pan-Immune–Inflammation Value (PIV) are powerful composite biomarkers reflecting the integrated effects of systemic inflammation, immune dysregulation, and nutritional status in patients with ischemic and hemorrhagic stroke. Both indices, easily derived from routine laboratory tests, exhibited substantial discriminative and prognostic value. The CALLY index, in particular, independently predicted stroke subtype and in-hospital mortality among hemorrhagic cases, emphasizing the clinical relevance of the inflammation–nutrition–immunity axis.

The differential performance of these indices across stroke subtypes likely reflects distinct immune activation pathways, where elevated PIV indicates heightened innate immune activity and reduced CALLY suggests impaired adaptive immunity and malnutrition. Their combined application could therefore enhance early risk stratification and guide precision-oriented treatment strategies.

Given their accessibility and low cost, CALLY and PIV may be integrated into electronic clinical decision-support systems for rapid prognostic evaluation. Future multicenter, prospective, and mechanistic studies are warranted to validate their prognostic accuracy, define standardized thresholds, and explore their interplay with molecular and cytokine-based markers to deepen understanding of stroke immunopathophysiology.

Among all indices evaluated, CALLY emerged as the strongest predictor, particularly in hemorrhagic stroke, underscoring the central role of the inflammation–nutrition–immunity axis. Future prospective and multicenter studies are required to validate these findings and to determine clinically applicable thresholds.

### 5.1 Limitations

This study has several limitations. First, its retrospective and single-center design restricts causal inference and may limit generalizability. Second, only baseline laboratory data obtained within 24 h of admission were analyzed; therefore, dynamic changes in inflammatory and nutritional indices could not be assessed. This single-time-point design may not fully capture the temporal dynamics of the inflammatory response. Third, unmeasured factors such as stroke severity, treatment differences, or subclinical conditions might have influenced the results. Finally, multicenter prospective studies are warranted to validate these findings and confirm their clinical applicability.

Another limitation of this retrospective study is that standardized clinical severity and functional outcome measures such as NIHSS and mRS were not consistently recorded in the historical electronic database, preventing their inclusion in the analyses. Similarly, imaging-based severity characteristics such as hematoma volume, ASPECTS, midline shift, or edema grade were not consistently documented in the radiology archive and therefore could not be incorporated into the analyses. Additionally, because the study cohort was generated through an electronic query that retrieved only cases with complete laboratory parameters required for index calculations, patients with missing key data were not captured as a separate group. Therefore, the exact number of potentially eligible but excluded patients could not be quantified retrospectively, which may introduce selection bias. Future prospective and multicenter studies with systematic data collection are needed to validate these findings and to enable the integration of inflammatory indices such as CALLY and PIV with established clinical scoring systems (e.g., NIHSS, mRS) through multivariable prediction models or nomogram-based clinical tools.

Because incomplete records were not captured as a separate group in the electronic database, the exact number of excluded patients could not be determined, preventing the construction of a detailed flow diagram.

## Data Availability

The raw data supporting the conclusions of this article will be made available by the authors, without undue reservation.
